# Neuroimmune Pathophysiology in Asthma

**DOI:** 10.3389/fcell.2021.663535

**Published:** 2021-05-13

**Authors:** Gandhi F. Pavón-Romero, Nancy Haydée Serrano-Pérez, Lizbeth García-Sánchez, Fernando Ramírez-Jiménez, Luis M. Terán

**Affiliations:** Department of Immunogenetics and Allergy, Instituto Nacional Enfermedades Respiratorias Ismael Cosío Villegas, Mexico City, Mexico

**Keywords:** asthma, neurotransmitters, neuropeptides, allergy, immunology

## Abstract

Asthma is a chronic inflammation of lower airway disease, characterized by bronchial hyperresponsiveness. Type I hypersensitivity underlies all atopic diseases including allergic asthma. However, the role of neurotransmitters (NT) and neuropeptides (NP) in this disease has been less explored in comparison with inflammatory mechanisms. Indeed, the airway epithelium contains pulmonary neuroendocrine cells filled with neurotransmitters (serotonin and GABA) and neuropeptides (substance P[SP], neurokinin A [NKA], vasoactive intestinal peptide [VIP], Calcitonin-gene related peptide [CGRP], and orphanins-[N/OFQ]), which are released after allergen exposure. Likewise, the autonomic airway fibers produce acetylcholine (ACh) and the neuropeptide Y(NPY). These NT/NP differ in their effects; SP, NKA, and serotonin exert pro-inflammatory effects, whereas VIP, N/OFQ, and GABA show anti-inflammatory activity. However, CGPR and ACh have dual effects. For example, the ACh-M3 axis induces goblet cell metaplasia, extracellular matrix deposition, and bronchoconstriction; the CGRP-RAMP1 axis enhances Th2 and Th9 responses; and the SP-NK1R axis promotes the synthesis of chemokines in eosinophils, mast cells, and neutrophils. In contrast, the ACh-α7nAChR axis in ILC2 diminishes the synthesis of TNF-α, IL-1, and IL-6, attenuating lung inflammation whereas, VIP-VPAC1, N/OFQ-NOP axes cause bronchodilation and anti-inflammatory effects. Some NT/NP as 5-HT and NKA could be used as biomarkers to monitor asthma patients. In fact, the asthma treatment based on inhaled corticosteroids and anticholinergics blocks M3 and TRPV1 receptors. Moreover, the administration of experimental agents such as NK1R/NK2R antagonists and exogenous VIP decrease inflammatory mediators, suggesting that regulating the effects of NT/NP represents a potential novel approach for the treatment of asthma.

## Introduction

Asthma is a disease characterized by chronic airway inflammation, leading to intermittent symptoms including wheezing, dyspnea, cough, and chest tightness, in combination with variable expiratory airway obstruction. It is estimated that 334 million people suffer from this disease worldwide (Enilari and Sinha, 2019). Asthma is caused by complex interactions between the environment and genetic factors, resulting in heterogeneity in clinical presentation, inflammation, and a possible remodeling of the airways (Tyler and Bunyavanich, 2019). Type I hypersensitivity (TIHS) is responsible for the greatest part of its pathophysiology (Kubo, 2017). However, the role of neurotransmitters (NT) and/or neuropeptides (NP) in this disease has been less explored than its inflammatory mechanisms.

Recently, anticholinergic drugs prescribed in chronic obstructive pulmonary disease (COPD) (Global Initiative for Asthma, 2020) have shown clinical efficacy in asthma when they are used as adjuvants (Novelli et al., 2012). Likewise, some experimental drugs that modulate the NT and NP response (Milara et al., 2013) have been proposed as therapeutic targets due to their physio-pathological actions in asthma. In this general review, we explain the interaction of both NT and NP with the immune system and bronchial environment in asthma, and their potential use as biomarkers and diagnostic tools, as well as their therapeutic use in patients with this disease in the future.

## Asthma Pathophysiology

A strategy used to classify asthma pathophysiology, taking into account its immunological heterogeneity, is the identification of inflammatory cellularity in fluids extracted from the airway (sputum or Bronchoalveolar lavage fluid -BALF-). There are four different groups according to the evidence provided by the cytology of the local samples: (Enilari and Sinha, 2019) eosinophilic, (Tyler and Bunyavanich, 2019) neutrophilic, (Kubo, 2017) mixed, and (Global Initiative for Asthma, 2020) paucigranulocytic. Eosinophilic inflammation is the main type of cellularity identified and is a consequence of allergic and non-allergic processes (Simpson et al., 2006). Allergy is associated with almost 60% of childhood and adult asthma, but is not the only condition that causes eosinophilic inflammation (Pearce et al., 1999; Simpson et al., 2006).

**FIGURE 1:**
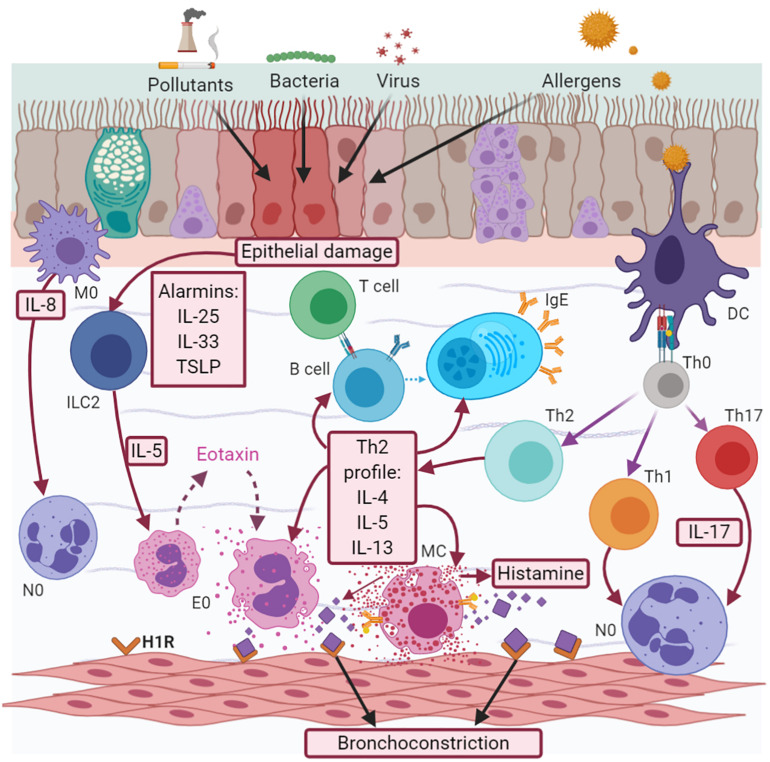
Asthma pathophysiology. Asthma is a complex interaction between cells, cytokines and chemokines. There are two effector cell responses, neutrophilic, and eosinophilic. The eosinophilic response is the most frequent, mediated by the synthesis of a Th2 cytokine profile (IL-5, IL-4, and IL-13) and histamine released when there is an IgE-mediated allergic response; or by alarmins (TSLP, IL-25, and IL-33) in the case of a non-allergic one. Neutrophilic response is less understood and maybe a transition from early Th2 or be a consequence of early Th1/Th17 secondary to macrophage activation and IL-8 release.

In allergic eosinophilic asthma, atopic subjects are predisposed to develop IgE-mediated allergic sensitization (atopy). Dendritic cells (DCs) take up the allergens (pollens, dust, and mold, among others), which are processed by endosomes and presented to T helper (Th) 2 lymphocytes (Th2), inducing the synthesis of a Th2 profile of interleukins (IL), such as IL-5, IL-4, and IL-13. IL-5 induces the maturation and survival of eosinophils. These cells migrate to the bronchial epithelium via chemoattractant factors, such as eotaxins (CCL11, CCL24, CCL26, and CCL5) (Teran, 2000; Rojas-Ramos et al., 2003) coupled to the CCR3 receptor, while IL-4 and IL-3 favor the change of immunoglobulin (Ig) isotype in B cells, with the subsequent production of IgE (Figure 1).

This immunoglobulin recognizes two types of receptors: high-affinity receptors (FcεRI) and low-affinity receptors (FcεRII or CD23). FcεRI receptors are expressed in mast cells (MCs), basophils, DCs, and eosinophils, but are also present in other cells, such as airway smooth muscle cells (ASM), epithelial, and endothelial cells. The coupling between IgE and FCεRI receptors in DCs amplifies their ability to present antigens, and in turn, the activation of allergen-specific Th2 cells is associated with the amplification of allergen-specific IgE production in a vicious cycle of the pathogenic mechanisms of allergic asthma. IgE acts in airway epithelial cells through the CD23 receptor, which is involved in the transport of IgE-allergen complexes across the polarized airway mucosal barrier (Matucci et al., 2018).

Activated eosinophils release mediators, such as major basic protein (MBP), reactive oxygen species (ROS), granulocyte-macrophage colony-stimulating factor (GM-CSF), IL-8, lipid mediators (cystenil leukotrienes -LTs), and histamine. MBP can mediate epithelial cell damage, while cysLTs contribute to airway remodeling. Neutrophil recruitment is induced by IL-8, whose expression is upregulated in the airways of patients with severe asthma and mixed cellularity (Nakagome and Nagata, 2018). Histamine is also released by basophils and MCs, and is associated with the induction of bronchial smooth muscle contraction, epithelial barrier dysfunction, and the increased secretion of mucus via the H1 receptor, while its coupling with the H2 receptor increases the capillary permeability (Yamauchi and Ogasawara, 2019). Eosinophils and MCs also play a relevant role, producing cysLTS and prostaglandin D2 (PGD2). This last mediator induces eosinophil chemotaxis through a chemoattractant receptor-homologous molecule expressed on TH2 cells (CRTH2), expressed in eosinophils, basophils, Th2 cells, and innate lymphoid cell type 2 (ILC2) (Yamauchi and Ogasawara, 2019). In non-allergic eosinophilic asthma, when the epithelium is injured by any factor, it synthesizes alarmines (IL-25, IL-33, and thymic stromal lymphopoietin-TSLP), which stimulate ILC2, leading to the synthesis of a Th2 profile without allergen-specific IgE involvement, which does not require antigen processing (Figure 1) (Kaur and Chupp, 2019).

The pathophysiology of non-eosinophilic asthma is not yet understood. Neutrophil cellularity is associated with both Th1 and Th17 interleukin profiles, as well as with the subsequent activation of macrophages and the release of neutrophil chemokines, such as IL-8 (Figure 1; Paplińska-Goryca et al., 2018). Evidence shows that the interaction between specific allergens and IgE/FcεRI on the neutrophil surface enhances functional responses by increasing the secretion of neutrophil products, such as matrix metalloproteinase 9 (MMP-9), neutrophil elastase (NE), myeloperoxidase, IL-8, and ROS (Radermecker et al., 2018).

Asthma treatment is based on corticosteroids (inhaled or oral), leukotriene antagonists and/or β-adrenergic agonists. Recently, the use of anticholinergics and biological antibodies (anti-IgE/anti-IL5) was approved depending on the severity of this disease; the use of any combination reduces inflammatory biomarkers and improves the symptoms (Global Initiative for Asthma, 2020).

## Bronchial Airway

### Airway Epithelium and PNEC

**FIGURE 2:**
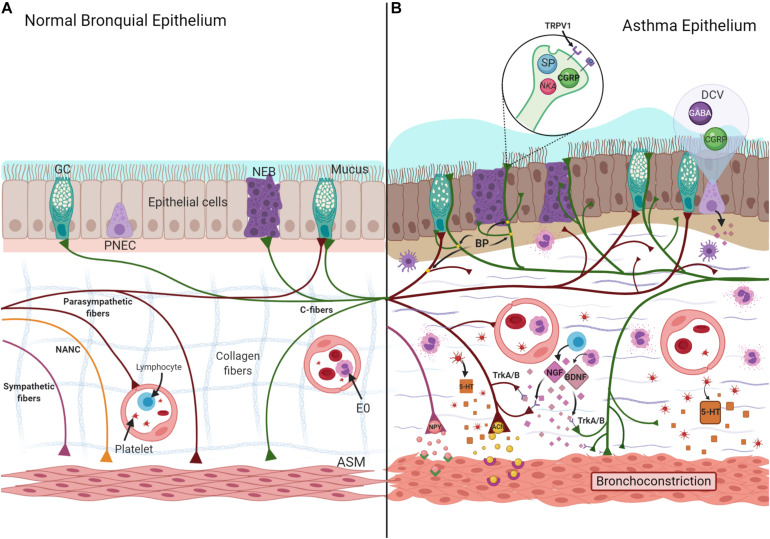
Normal **(A)** and asthma-induced epithelial damage **(B)**. Neurotrophins (NGF- Nerve Growth Factor and BDNF-Brain-Derived Neurotrophic Factor) are synthesized by eosinophils (E0) and lymphocytes, the union to their receptor (TrkA/B), localized in nerves induce a greater length of nerves, increasing branch points (BP), and exposition of nerve endings to the lumen. NT and NP (SP, CGRP, NKA, ACh, 5-HT) are stored and released from these TRPV1 + fibers, increasing mucus secretion, collagenous deposition, and ASM hyperplasia, characteristic findings associated to asthma. Likewise, 5-HT and NPY increase the bronchoconstriction induced by ACh. ASM, airway smooth muscle; DVC, dense core vesicles; GC, Goblet cell; NANC, non-adrenergic/non-cholinergic; NEB, neuroepithelial bodies; PNEC, pulmonary neuroendocrine cell; TRPV1, transient receptor potential vanilloid 1.

The bronchial epithelium is a pseudostratified ciliated columnar epithelium with goblet cells (Rock et al., 2010). However, there are other cells, such as pulmonary neuroendocrine cells (PNECs) that constitute approximately 1% of the airway mucosa. PNECs are short pyramid cells with cytoplasmic projections to the lumen grouped in mini-clusters (five cells) or neuroepithelial bodies (NEB) (>20 cells), located at branch junctions (Kuo and Krasnow, 2015). These cells contain NP, NT, and amines stored in dense-core vesicles (DCV) (Branchfield et al., 2016). The vagal fibers of the autonomic nervous system (ANS) comprise the majority of bronchial airway innervation (Kistemaker and Prakash, 2019). However, the mechanism underlying the interaction between PNEC and ANS has yet to be well described. Indeed, some studies have reported that only the NEB are innervated, and not the PNEC (Figure 2; Brouns et al., 2006; Kuo and Krasnow, 2015).

### Innervation

Two types of nerve fibers (A and C) provide afferent innervation. Type A fibers are myelinated axons classified according to their diameter and conduction impulse. For example, Aδ fibers have a smaller diameter and a slower conduction than Aα fibers. They are considered as mechanoreceptors, and their activation depends on the deep and breathing rate (Nassenstein et al., 2018). In contrast, type C fibers are thin unmyelinic axons with slower conduction velocities than A fibers (Feldman et al., 2017), located in the glands, microvasculature, ASM, and NEB (Drake et al., 2018), and are classified as chemoreceptors and nociceptors (Feldman et al., 2017), transmitting afferent impulses when reacting with stimuli, such as changes in temperature, pH, and mediators released by tissue damage and inflammation (Narula et al., 2014), inducing reflex responses that include mucus discharge, bronchoconstriction, and cough (Undem and Carr, 2002). C fibers have specific receptors for NP, such as Substance P (SP), Neurokinin A (NKA), and Calcitonin Gene-Related Peptide (CGRP), which are involved in ASM contraction (Nassenstein et al., 2018).

Afferent vagus fibers transmit impulses from the airway to the jugular ganglia (branch of the superior laryngeal nerve) and nodose ganglia (branch of the recurrent laryngeal nerve) (Undem et al., 2004), subsequently traveling to the caudal nucleus of the solitary tract (Freeman et al., 2017). By contrast, efferent innervation is comprised by ANS and non-adrenergic non-cholinergic nerves (NANC). The NANC system shares the parasympathetic nerves derived from the dorsal motor nucleus of the vagus (Kistemaker and Prakash, 2019). On the other hand, sympathetic fibers come from the intermediolateral nucleus (T2 to T7 segments). Acetylcholine (ACh) mediates the physiological actions of the parasympathetic system, which induces bronchoconstriction. However, NANC modulates these actions using diverse NP. For example, vasoactive intestinal peptide (VIP) induces bronchodilation. Likewise, sympathetic fibers interact with the airway through epinephrine, exerting the same effect.

### Neuronal Remodeling in the Epithelium

Biopsies from moderate-intermittent asthma patients have a greater length, nerve branching, and more branch points than patients with mild asthma and controls (Drake et al., 2018), exposing the nerve endings to the bronchial lumen, leading to neuronal remodeling (Ollerenshaw et al., 1991). This process has two phases: a regenerative phase, during which axons undergo regrowth and dendrites become new connections, and a degenerative phase, characterized by the incorporation of neurites and synapses (Figure 2; Alyagor et al., 2018).

Neurotrophins, such as nerve growth factor (NGF) and brain-derived neurotrophic factor (BDNF), are synthesized by neurons of the central nervous system (CNS) and ANS as small active peptides that, upon coupling with their receptors (tropomyosin receptor kinase A and B), play a substantial role in neuronal remodeling (Keefe et al., 2017). However, bronchial epithelial cells, ASM (Ricci et al., 2004), immune cells, such as lymphocytes (Ehrhard et al., 1993), or eosinophils (Kobayashi et al., 2002), and PNEC can synthesize them. In fact, early life allergen exposure appears to elevate the level of neurotrophins and cause PNEC hyper-innervation and nodose neuron hyperactivity, inducing mucin secretion (Barrios et al., 2017).

With regards to airway remodeling, TNF-α increases the synthesis of BDNF from ASM and enhances the production and deposition of collagen-1, collagen-3, and fibronectin, as well as the activity of MMP-2 and MMP-9 (Freeman et al., 2017), and is involved in muscle cell proliferation (Aravamudan et al., 2012). It has been described that collagen-I favors the expression of CCL5, GM-CSF, and exotoxin (Peng et al., 2005; Chan et al., 2006), contributing to persistent inflammation (Burgess, 2009). NGF has been found to exert similar actions on the components of the extracellular matrix (Huang et al., 2015).

Chronic inflammation caused by allergen sensitization induces the synthesis of new receptors at the nerve fibers. For example, type A fibers can express another receptor as transient receptor potential vanilloid 1 (TRPV1) (Bron et al., 2003), an ionic channel of transient release potential mainly localized in C fibers (Nassenstein et al., 2018), whose expression is modulated by NGF and BDNF (Bron et al., 2003; Figure 2).

TRPV1 is activated by a wide range of stimuli, such as high temperature, protons, voltage (Banner et al., 2011), or endogenous inflammatory factors, such as arachidonic acid metabolites (Hwang et al., 2000). The activation of TRPV1 induces a reflex response, such as cough and bronchoconstriction (Bhattacharya et al., 2007). This receptor is increased in patients with asthma compared to controls and patients with mild asthma (McGarvey et al., 2014).

Capsaicin, a molecule with pungent properties contained in some foods, such as chili, has been used to evaluate the functions of TRPV1 (Groneberg et al., 2004). It is a simple, safe, and reproducible cough provocation test. This challenge is applied in the algorithm of idiopathic chronic cough (Morice et al., 2007) and is a useful tool to evaluate the efficacy of asthma treatment. For example, the use of inhaled corticosteroids (ICS) for at least 3 months reduces cough induced by capsaicin (Di Franco et al., 2001; Ekstrand et al., 2011). Besides, in cough variant asthma, the capsaicin challenge predicts the ICS treatment response better than the methacholine challenge (Park et al., 2007).

Some reports have indicated that the use of anticholinergic agents, such as tiotropium, improves refractory cough in asthma patients and augments the threshold to this substance during the challenge. This suggests that tiotropium suppresses the neuronal activity of TRPV1, a mechanism independent of the muscarinic type 3 (M3) receptor blockade (Fukumitsu et al., 2018).

## Acetylcholine

Acetylcholine (ACh) is one of the main neurotransmitters both in CNS and peripheral nervous system (PNS) (Vogt, 2018). Its release via exocytosis from the parasympathetic nerve endings to the intercellular space. In 1963, ACh was found to be produced in non-nerve cells (Wessler and Kirkpatrick, 2001) including immune cells (Kawashima et al., 1998; Fujii et al., 2012) giving rise to different responses depending on the stimulated receptor (Pedersen et al., 2018). One of the principal receptors where it exerts its function is the muscarinic ACh receptors (mAChRs), belonging to the family of G protein-coupled receptors (GPCRs), with which they share a high degree of homology. Five types have been described (M1–M5) (Caulfield and Birdsall, 1998), three of which exert physiological effects in the airways, namely M1, M2, and M3. M1 is localized over the alveolar walls, M2 in ASM, and M3 in airway epithelium, ASM, and submucosal glands (Mak et al., 1992).

In murine models of allergic asthma (MMAA), ACh contributes to allergen-induced remodeling mainly through the M3 receptor, but not through the M1 or M2 receptors, increasing the mass of ASM (Kistemaker et al., 2014). Likewise, mAChRs are involved in IL-8 synthesis by these cells, enhancing inflammation (Oenema et al., 2010). The agonists of ACh are related to the modulation of a specific type of mucin known as MUC5AC (Kistemaker and Gosens, 2015), the main mucin glycoprotein responsible for mucus viscoelasticity in asthma (Kirkham et al., 2002; Morcillo and Cortijo, 2006). Additionally, ACh induces collagen synthesis (Haag et al., 2008) via M2 and M3 localized in fibroblasts (Matthiesen et al., 2006) and increases its thickness upon stimulation with TGF-β (Grainge et al., 2011). This mechanism plays a role in the process of pro-fibrotic airway remodeling (Haag et al., 2008). However, the use of anticholinergic drugs, such as tiotropium bromide in chronic models of asthma, reduces M3 expression in bronchia, the Th2 profile, and airway hyperresponsiveness (AHR) (Kang et al., 2012; Kurai et al., 2018).

There is evidence that ACh induces a range of effects on immune cells. For example, lung macrophages express all the components from ACh synthesis, including M1-M5 receptors. The ACh agonist stimulates the production of *de novo* mediators, such as leukotriene B4 (LTB4) via M2 and M3, where the antagonist for the latter receptor inhibits this process (Koarai et al., 2012). Likewise, the content of eosinophilic granules, such as eosinophil peroxidase (EPO), increases the expression of *ChAT* and *VAChT* genes (necessary for the synthesis and storage of ACh) in fibroblasts. However, other eosinophilic mediators, such as MBP or eosinophil-derived neurotoxin (EDN), do not have this effect (Akasheh et al., 2014). In DCs treated with ACh, this NT stimulates the expression of the Th2−promoter OX40L, the production of the Th2−chemokines, such as CCL22 or CCL17, and a Th2 profile with reduced IFN-γ synthesis, suggesting that ACh can further promote a Th2 response even in the presence of a strong Th2 inducer, such as TSLP (Gori et al., 2017).

Inflammation mediated by lipopolysaccharide (LPS) and IFN-γ induces M3 expression and fibroblast proliferation (Español et al., 2014). COPD treatment based on anticholinergic drugs, such as Aclidinium, blocks the transduction of M1, M2, and M3 receptors (Milara et al., 2013), inhibiting the development of these cells and collagenous deposition (Milara et al., 2012), similar to the effect that occurs in asthma.

**FIGURE 3:**
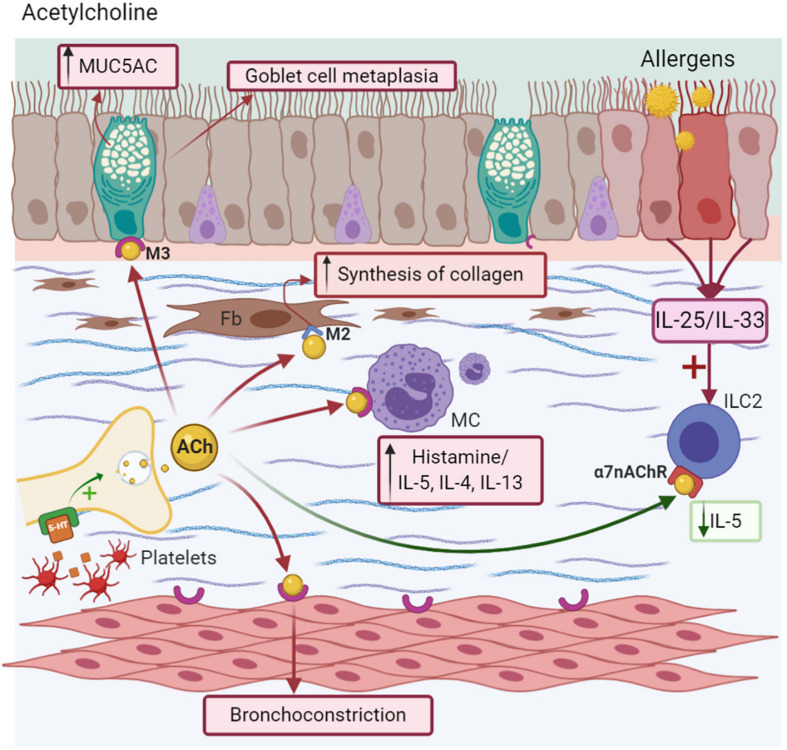
Acetylcholine-ACh. Nerve endings release ACh after depolarization or 5-HT stimuli, causing goblet cell metaplasia, MUC5AC secretion, and bronchoconstriction. It induces Mast cells (MC) degranulation via the M3 receptor and collagen deposition in fibroblast (Fb) via the M2 receptor. Anti-inflammatory effects as decreasing of IL-5 synthesis are due to the α7nAChR activation in ICL2.

Other receptors stimulated by ACh include nicotinic receptors (nAChR), which are proteins that are ligand-gated ion channels and localized near to the parasympathetic ganglia, where they facilitate neurotransmission (Racké et al., 2006). Specifically, the alpha 7 nicotinic acetylcholine receptor (α7nAChR) is expressed on macrophages and neutrophils, playing an essential role in attenuating the inflammatory response by stimulating the vagus nerve during systemic inflammation (Wang et al., 2003). Its activation induces the suppression of NF-kB with the subsequent inhibition of pro-inflammatory cytokines (TNF-α, IL-1, and IL-6) and chemokines from inflammatory cells in alveolar macrophages, resulting in the attenuation of lung inflammation and injury (Wang et al., 2003; Li J. et al., 2011). ILC2 express α7nAChR, which attenuates the expression of NF-kB and GATA-3, reducing the cytokine production of IL-5 and IL-13. Likewise, it modulates IL-33, which is necessary for activating this kind of lymphocyte (Galle-Treger et al., 2016). Additionally, the high expression of α7nAChR in the adrenal medulla is associated with the release of endogenous epinephrine in MMAA, helping to resolve AHR (Figure 3; Chen et al., 2017).

The ACh agonist, methacholine, has been used for the diagnosis of asthma (methacholine challenge). Among the main indications are staging the degree of severity AHR and evaluating the effectiveness of the medication in acute and chronic states, or any change in the therapeutic modality (Crapo et al., 2000; Global Initiative for Asthma, 2020). Among all the possible therapies involving both NT and NP, the use of an ACh antagonist is the only therapy approved for the control of asthma (Global Initiative for Asthma, 2020). The effects of muscarinic antagonists include the prevention of both mucous gland hypertrophy and allergen-induced goblet cell hyperplasia, an effect similar to ICS, and partially, the reduction of eosinophilia in the submucosal compartments of cartilaginous and non-cartilaginous airway areas in animals challenged with ovalbumin (OVA) (Bos et al., 2007). Moreover, in a chronic model murine of asthma (MMA) (Kistemaker et al., 2016), muscarinic antagonists were found to decrease smooth muscle mass in addition to ICS, low cell counts (macrophages, eosinophils, and lymphocytes), and decreased IL-5 levels in BALF than other Th2-profile cytokines. Likewise, mice treated with tiotropium had a smaller area of expression of collagen type I and III and significantly reduced M3 receptor expression (Kang et al., 2012). The addition of tiotropium and other antagonizes of M2 and M3 used in COPD as Aclidinium or Glycopyrronium, improves the lung function, reduced the need of oral steroids, and provides beneficial effects on symptom control in patients of all ages with severe asthma, not controlled with convectional therapies (Matera et al., 2020).

## Substance P

Substance P (SP) is a member of the Tachykinins (TAC) family. This NP is present in both the CNS and PNS (Lai et al., 2008), and in conjunction with CGRP and VIP, mediates the NANC system. Voedisch et al. (2012) TRPV1 + sensory nerves produce and store SP in the large-DCV. This NP is not only released from these neurons upon allergen stimulus (He et al., 2019; Perner et al., 2020), it can also be synthesized by non-neuronal cells, such as lymphocytes (Morelli et al., 2020), DC, eosinophils (Lambrecht et al., 1999), and macrophages (Ho et al., 1997). Once exocytosed from the neuronal soma or axonal terminals, it couples to its specific receptor (Neurokinin receptors -NKRs-), belongs to the GPCR family (Badri and Smith, 2019), expressed either on the same cell or on the neighboring cells (epithelial, endothelial, ASM cells, fibroblasts, and immune cells). NK1R and its isoforms, namely NK1R-F and NK1R-T (Blum et al., 2008), have a higher affinity than NK2R and NK3R (Schelfhout et al., 2006). Some immune cells, such as Th1, Th17, DC, and neutrophils, express NK1R (Serra et al., 1988; Marriott and Bost, 2001; Morelli et al., 2020). However, eosinophils have NK2R (Raap et al., 2015). Specifically, NK1R-F is expressed in the human brain, while NK1R-T is expressed in the CNS and peripheral tissues, such as bronchial vessels, epithelium, submucosal glands, or endothelium, and is related to inflammation (Mapp et al., 2000; Caberlotto et al., 2003). Both NK1Rs and NK2Rs are found in bronchial ASM cells (Nederpelt et al., 2016) and may mediate bronchoconstriction (Maghni et al., 2003).

**FIGURE 4:**
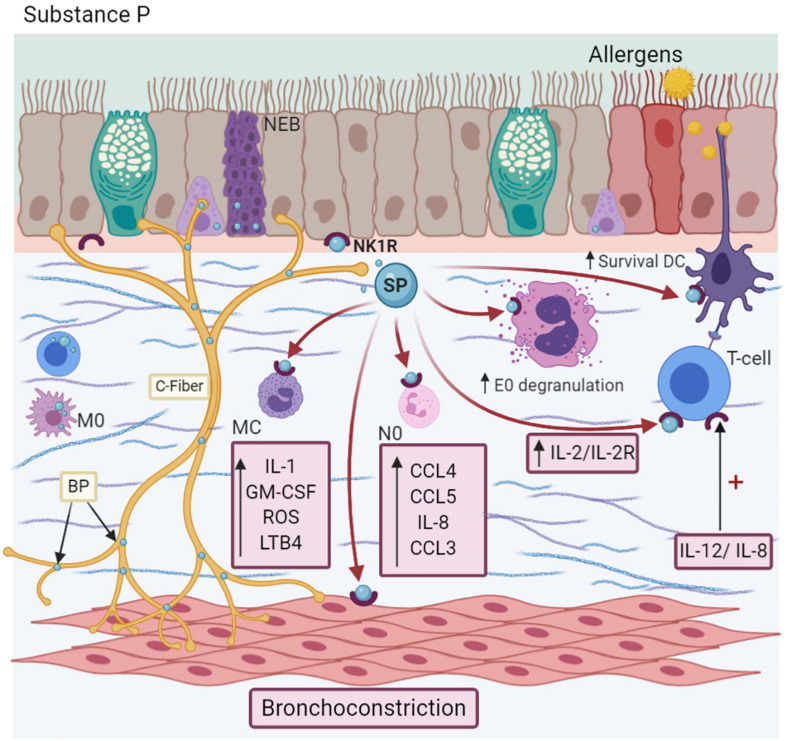
Substance P-SP. C-fibers, NEBs, T cells, and macrophages (M0) synthesize SP. SP induces the synthesis of IL-1, GM-CSF, ROS, and LTB4 synthesis in Mast cells (MC); CCL4, CCL5, IL-8, and CCL3 production in neutrophils (N0); degranulation of eosinophils (E0), dendritic cells (DCs) survival and the promotion of Th2 differentiation. Besides, it causes bronchoconstriction by decreasing SERCA/NCX expression. Its NK1R receptor is localized in many cells and is upregulated in an IL-12/IL-8 microenvironment.

SP is present in the serum and BALF of allergic asthma patients (Nieber et al., 1993). In fact, the bronchial branch points are associated with greater SP expression in patients with moderate persistent asthma (Drake et al., 2018). SP induces chemokine synthesis, such as CCL4, CCL5, and IL-8 (Spitsin et al., 2017) specifically, and modulates the chemotaxis of neutrophils, inducing the expression of CXCL2 and CCL3 (Sun et al., 2007). It is also involved in the migration of basophils and eosinophils, an effect comparable to other chemotactic agents, such as LPS (Cima et al., 2010) or C5a, respectively, in an IL-3 microenvironment (Raap et al., 2015). The use of a selective NK1R antagonist (*L733,060*) interferes with this mechanism (Morelli et al., 2020). In immune cells, SP induces T-lymphocyte proliferation *in vitro* by IL-2/IL-2Ra synthesis (Kulka et al., 2008), while in MC, it releases IL-1, GM-CSF, chemokines, oxygen radicals, and LTB4 (Kulka et al., 2008; Li et al., 2018). In addition to IL-33, SP secretes IL-31, TNF-α, and vascular endothelial-derived growth factor (Figure 4; Taracanova et al., 2017; Petra et al., 2018). Likewise, SP activates neutrophils through the expression of adhesion molecules, such as CD11b integrin (Sun et al., 2007), in DC, enhancing their survival, which is indispensable for maintaining the eosinophilic airway inflammation perpetuating the Th2 response characteristic of asthma (Voedisch et al., 2012). On the other hand, it downregulates the FcεRI expression in MC (McCary et al., 2010). In turn, interleukins modulate the effects of this NP. For example, IL-12 and IL-18 induce NK1R expression in T cells (Weinstock et al., 2003), but IL-12 and IL-23 enhance *TAC1* expression in macrophages (Blum et al., 2008). However, IL-10 and TGF-β play a relevant role in downregulating these effects (Blum et al., 2008).

SP it has been identified in asthma patients, even is more frequent than in patients with gastroesophageal reflux (GER) (Emilsson et al., 2016). During the bronchoconstriction process, SP increases intracellular Ca^2+^ in ASM cells by decreasing the sarco/endoplasmic reticulum Ca^2+^-ATPase (SERCA) and Na*^+^*/Ca^2+^ exchanger (NCX) proteins, augmenting the availability of calcium for contraction (Mahn et al., 2009; Li and Shang, 2019). The administration of NK1R antagonists, such as *WIN62577* or *GR304050*, increases SERCA protein with a subsequent decrease in Ca^2+^ concentration, a similar effect of *KB-R7943*, an NCX-specific inhibitor (Li M. et al., 2011; Li and Shang, 2019). In a similar context, the use of an experimental antagonist of NK1R (*CP96345*) (Yaraee and Ghazanfari, 2009) was found to reduce the TGF-β levels, favor ASM relaxation, and reduce the impact of fibrosis on airway remodeling (Li and Shang, 2019).

Additionally, experimental antagonists for this NP (Aprepitant) have shown to improve cough, a cardinal symptom of asthma, in cancer patients, so they can be considered to treat conditions such as asthma (Noronha et al., 2021). However, the drugs designed to block SP in asthma have had limited efficacy in clinical trials, possibly due to unanticipated changes in SP signaling occurred in asthma or changes in its metabolism (Drake et al., 2018).

## Tachykinins

The TAC family comprises NKA, neurokinin B (NKB), Hemokinins (HKs) 1–4, Endokinins (EKs) A-D (Helyes et al., 2010), Neuropeptide K, and Neuropeptide γ, in addition to SP. TACs receptors (NK1R, NK2R, and NKR3) belong to the GPCR family. NKA and NKB bind specifically to NK2R and NK3R, respectively (Nederpelt et al., 2016), whereas HK-1, EKs, and SP bind to NK1R (Kurtz et al., 2002; Grassin-Delyle et al., 2010). However, SP, NKA, and NKB are able to couple to all receptors (Schelfhout et al., 2006; Nederpelt et al., 2016). The modulation of TAC is mediated by NEP (Neprilysin or Enkephalinase). Its deficiency is related to mucus hypersecretion, vascular hyperpermeability, and inflammation in human lung biopsies (Baraniuk et al., 1995).

In OVA-sensitized mice, sensory nerve endings release NKA and SP, followed by an increased temperature, enhancing the percentage and diameter of TAC-immunoreactive neurons, identified as TRPV1 + sensory neurons (Hsu et al., 2013; Le et al., 2014). A positive correlation between reflux and SP/NKA sputum levels was observed in asthma patients with gastroesophageal reflux disease (GERD), suggesting that the thermal or chemical mechanisms involved in GERD allows for the release these NPs (Patterson et al., 2007).

NKA and SP mainly modulate NANC excitatory responses in the airway (Kajekar and Myers, 2008). In human lung biopsies, the three receptors are localized in ASM (Mizuta et al., 2008a), suggesting their role in ASM contraction. In OVA-sensitized guinea pig models, bronchoconstriction induced by NKA, NKB, and SP in this order, are likely to be induced by ACh (Daoui et al., 2000). In the same context, HK-1, EKA, EKB, and the agonist of NKB (*[MePhe^7^]-NKB*) also induce bronchoconstriction *in vitro* in human lung biopsies, in contrast to EKC and EKD (Grassin-Delyle et al., 2010; Corboz et al., 2012).

On the other hand, in MMA mediated by the Th1 response, IFN-γ increases NK2R expression in ASM and the NKA levels in BALF, as well as inducing AHR in a dose-dependent manner. In deficient-STAT1 mice, these responses were absent (Kobayashi et al., 2012). These effects have been described in human DCs localized in lung and macrophages from asthma patients (Ohtake et al., 2015). The NKA-NK2R axis stimulates the synthesis of IFN-α and IFN-β in human DCs (Kitamura et al., 2012).

**FIGURE 5:**
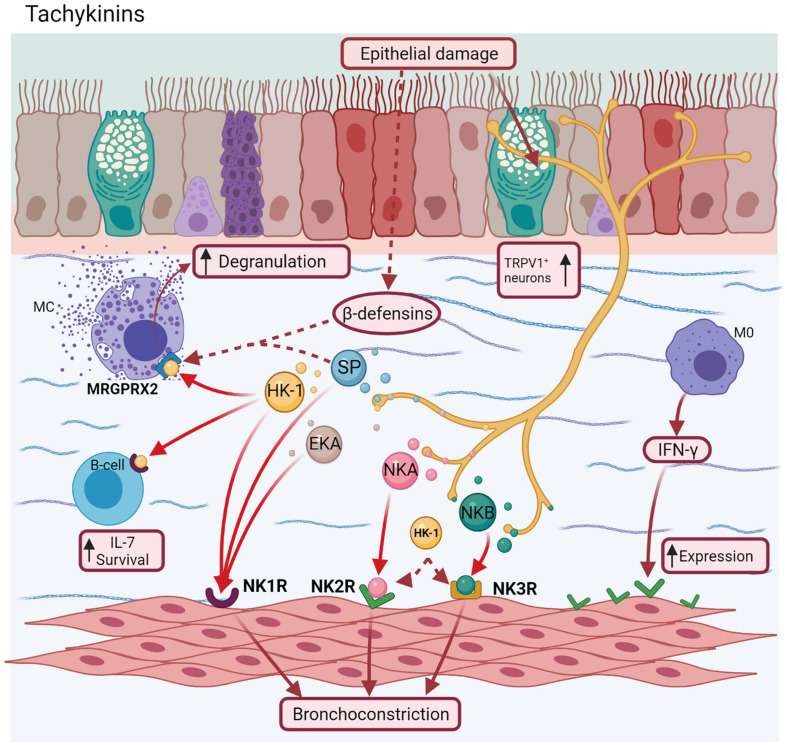
Tachykinins. Neurokinin A (NKA) and Neurokinin B (NKB) bind to NK2R and NK3R receptors, respectively, whereas Hemokinins (HK-1), Endokinins (EKA/B), and SP bind to NK1R. However, SP, NKA, and NKB can couple to all receptors, localized mainly in airway smooth muscle inducing airway bronchoconstriction. Additionally, HK-1 and other ligand as β-defensins, can join a novel receptor implicated in allergic asthma (MRGPRX2) of mast cells, favoring their degranulation.

There is scarce evidence of asthma about other TAC. Exists recent reports about the activation of HK-1 by Mas-related G-protein coupled receptor member X2 (MRGPRX2) (Manorak et al., 2018; Thapaliya et al., 2021). SP (Gaudenzio et al., 2016) and other ligands as β-defensins, a type of antimicrobial peptides (Guaní-Guerra et al., 2011) released after epithelial injuries (Subramanian et al., 2013), also activate this receptor. This fact was confirmed after a selective NK1R antagonist did not inhibit these effects (Manorak et al., 2018). MRGPRX2 could be also a promising serum biomarker in allergic asthma for monitoring treatment outcomes and determining personalized ICS dose. However, more studies are needed to establish this role (An et al., 2020). HK-1 is also involved in mouse pre-B cell survival and proliferation by increasing IL-7 levels, whereas the NK1R antagonist (*L732138)* increases apoptosis in these cells (Zhang et al., 2000; Figure 5).

Many antagonists of TAC receptors have been evaluated for their potential as biomarkers or pharmacological targets in asthma (Ramalho et al., 2011). In the first case, NKA levels in sputum were found to be correlated with asthma exacerbations in children, showing high levels of both NKA and eosinophil count even after remission, compared to the control group (Mostafa et al., 2008). On the other hand, the use of CS*-*003, a triple NKRs antagonist, administered by inhalation in patients with mild-to-moderate asthma, showed less bronchoconstriction in methacholine challenge. This effect had a duration of ∼8 h without any adverse effects (Schelfhout et al., 2006). Likewise, it was found to inhibit NKA/NKB-induced bronchoconstriction and SP-induced vascular hyperpermeability in guinea pigs (Nishi et al., 2000). The use of NK2R antagonists, such as *MEN-10376* and *SR48968*, reduced the lung insufflation pressure and abolished the effect of HK-1-induced bronchoconstriction, respectively (Krishnakumar et al., 2002), while the antagonists for NK3R (*SB223412* and *SR 142801)* reduced NKB-induced AHR and pulmonary inflation pressure (Corboz et al., 2012). The blockage of these effects by experimental drugs and others as concludes the role of TAC (NKA) as a necessary mediator in the bronchospasm (Joos et al., 2004).

## Calcitonin Gene-Related Peptide

**FIGURE 6:**
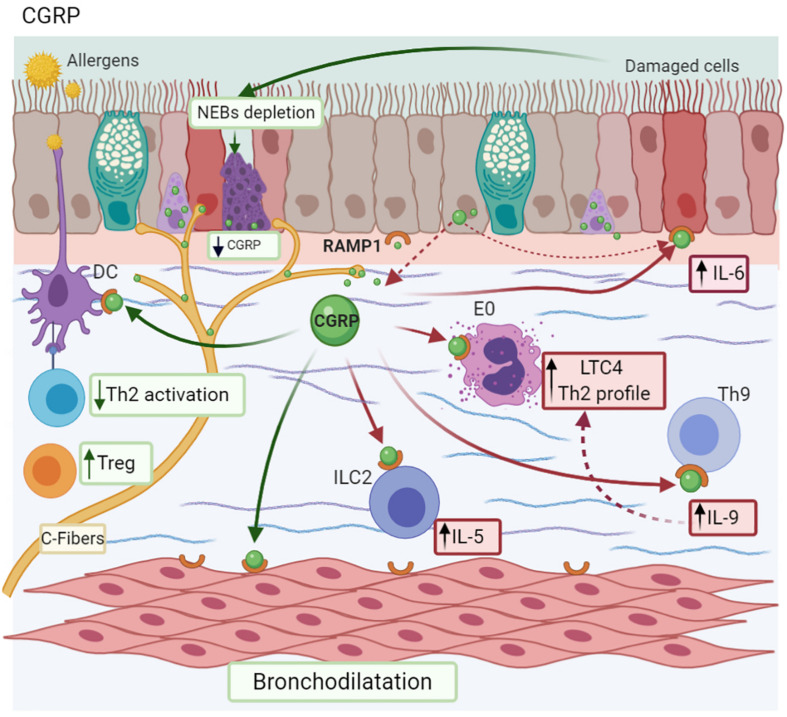
Calcitonin gene-related peptide-CGRP. CGRP is synthesized and stored in C-fibers, neuroepithelial bodies (NEBs), and the epithelium. CGRP promotes a Th9 profile after allergen exposure by RAMP1 activation; IL-6 secretion by the bronchial epithelium; and IL-5 synthesis by ILC2 cells. Additionally, epithelial damage provokes NEBs depletion with the subsequent decrease in CGRP levels. Depending on the context, it also causes bronchodilation and promotes Treg differentiation, reducing Th2 activation.

Calcitonin-gene related peptide is a NP present in two isoforms both in humans (I/II) and rats (α/β), which have similar homology (>90%) and biological activity (Russell et al., 2014). αCGRP is localized in both the CNS and PNS, whereas βCGRP is present in the enteric nervous system (Muddhrry et al., 1988) and immune cells (Xing et al., 2000), and is specifically synthesized in airways by PNEC (Sui et al., 2018). CGRP is co-stored with SP at the nerve ending of sensory neuron C fibers into the airways (Kajekar and Myers, 2008). Its receptor is a heterodimeric complex called Receptor Activity–Modifying Protein 1 (RAMP1) (McLatchie et al., 1998), which is expressed by airway epithelial cells (Li et al., 2014) and immune cells, such as Th9 cells (Mikami et al., 2013). In MMAA, DCs are localized next to vagal sensory neurons, where there is a CGRP neuron proliferation (Figure 6; Le et al., 2014).

The association between TRPV1 in lung tissues and an increase of CGRP in the BALF of OVA-sensitized mice has been described (Kim et al., 2020). The induction of the internalization of its receptor in airway epithelium and the subsequent expression of inflammatory interleukins, including IL-6, are among the effects of CGRP. Interestingly, biopsies from asthma patients support this observation, with reduced levels of RAMP1 compared to the controls (Bonner et al., 2010).

Calcitonin-gene related peptide modulates the Th9 response (response related to TIHS), inducing the expression of GATA3 and PU.1 (transcription factor of Th9 cells) and IL-9 production, enhancing airway inflammation (Mikami et al., 2013). Higher concentrations of CGRP could be released by CCL17 more than other inflammatory interleukins (IL-1, TNF-α, and IL-13) by a CCR4-dependent mechanism, which plays a role in the late asthmatic reaction. CCL17 may amplify the vascular component of the inflammatory response by stimulating epithelial cells to release CGRP (Bonner et al., 2013). This mechanism represents a possible therapeutic target for vascular events in patients with asthma and allergic inflammation (Bonner et al., 2013).

On the other hand, ILC2 express RAMP1. When the axis CGRP/RAMP1 interacts, it induces an increase in IL-5 production from these cells in an IL-25 and IL-33 microenvironment, inducing the maturation and activation of ILC2, but does not affect their proliferation. Similarly, CGRP is recruited to eosinophils and promotes the synthesis of leukotriene C4 triggering the Th2 response (Figure 6; Sui et al., 2018).

In allergen-induced late reactions, CGRP increases in both BALF and biopsies from allergic asthma patients after the inhalation of allergen-derived T-cell peptide epitopes, in comparison to SP and NKA levels, causing vasodilatation and edema (Kay et al., 2007). There is also evidence that this NP enhances the edema induced by histamine and SP (Brain and Williams, 1985). In addition, CGRP could exert other effects involved in asthma, such as AHR. In a rabbit model of ozone-induced AHR, CGRP stimulates an early inflammatory response that contributes to cleaning up of irritants (Ren et al., 2004).

Depending on the context, this NP has anti-inflammatory effects. For example, CGRP activates adenylate cyclase, which results in increased cellular levels of cyclic AMP, a pathway usually associated with bronchodilation (Dakhama et al., 2002). On the other hand, AHR induced by allergen exposure results in the depletion of NEB and submucosa plexus, followed by a decrease in CGRP. Interestingly, the exogenous administration of α-CGRP reduced both AHR and inflammation induced by eosinophils, comparable to anti-IL-5 antibody (Dakhama et al., 2002).

Calcitonin-gene related peptide inhibited DC maturation in mice lungs, followed by the decrease in antigen-specific T cell activation (specifically Th2) and the increase in Treg cells (Rochlitzer et al., 2011; Peng et al., 2018). Likewise, reduces the eosinophil counts and increases the levels of IL-10 in BALF (Rochlitzer et al., 2011). These mechanisms suggest that CGRP could also represent a new therapeutic target in asthma therapy, as an anti-inflammatory mediator.

## Serotonin

Serotonin (5-HT) is an NT and vasoactive amine that participates in numerous physiological processes. Intestinal enterochromaffin cells synthesize ∼90% of this NT (Arreola et al., 2015). However, is stored in dense granules of platelets 5-HT has seven receptor families (5-HT_1_-_7_), with their subtypes mainly associated with G proteins, except for 5-HT_3_, which is a ligand-controlled cation channel. Owing to the great variety of receptors and their extensive distribution, they are involved in a wide range of functions (Andrade et al., 2019).

Platelets are the main source of 5-HT in the lungs (Dürk et al., 2013). These cells are capable of active extravasation in this organ (Pitchford et al., 2008), where they release this NT (Dürk et al., 2013). Both processes promote platelet recruitment via the expression of *P*-selectin and its respective ligand (integrins), localized in eosinophils and lymphocytes, as observed in MMAA (Pitchford et al., 2005).

However, PNEC (Fu et al., 2002) and MCs are able to synthetize it (Kushnir-Sukhov et al., 2007). This effect increases in the presence of hypoxia and IL-33, respectively (Sjöberg et al., 2015). In mature DCs, 5-HT modulates the production of IL-1β and IL-8 through 5-HTR_3__/__4__/__7_ receptors (Idzko et al., 2004). A similar effect has been reported in peripheral mononuclear blood cells (PMBC) (Cloëz-Tayarani et al., 2003). In addition, NT increases the migration of pulmonary DCs to draining lymph nodes and induces the expression of a Th2 profile in these cells (Müller et al., 2009).

Additionally, ASM cells express 5-HT_2__*A/*__3__/__4__/__7_ (Fernandez-Rodriguez et al., 2010; Segura et al., 2010) in MMAA, which mediates bronchoconstriction (Arreola-Ramírez et al., 2013), activating its receptors on parasympathetic ACh-containing neurons, resulting in the release of ACh (Figure 4; Fernandez-Rodriguez et al., 2010). Interestingly, TNF-α up-regulates the contraction mediated by 5-HT via the 5-HT_2__*A*_ receptor (Adner et al., 2002). On the other hand, some reports have shown that patients with asthma showed increased levels of 5-HT in BALF compared to healthy control subjects (Dürk et al., 2013). Likewise, lung function was negatively correlated with an increase in 5-HT (Lechin et al., 1996). Consequently, the reduction of the plasma concentration of free 5-HT could be useful in the treatment of asthma patients. For example, there is an anecdotic report that evaluated the use of Tianeptine (an antidepressant), a drug that decreases plasma 5-HT by enhancing its reuptake. In a double-blind placebo control developed in patients with a weak response to conventional asthma treatment, this therapeutic approach was found to improve lung function and diminish symptoms in asthma patients (Lechin et al., 1998).

## Gamma-Amino Butyric Acid

Traditionally, Gamma-aminobutyric acid (GABA) exerts inhibitory neuronal functions (Xu et al., 2017). GABA is stored in vesicles and then released by exocytosis into the synaptic space. Its coupling to GABA receptors-GABARs (α/A, β/B, γ/C) and their subunits induces the opening of K^+^ ion channels to allow for the efflux of K^+^ and the influx of Cl^–^, resulting in hyperpolarization and a decrease in neuronal excitability (Sarasa et al., 2020). By contrast, GABA_*B*_R are GPCRs (Lu and Inman, 2009).

Epithelial cells express all the components for local GABA synthesis, release, and coupling with GABA_*A*_ and GABA_*B*_ receptors, creating an autocrine and/or paracrine system on airway epithelium and ASM (Mizuta et al., 2008b; Zaidi et al., 2011). In the epithelium, GABA exerts effects associated with bronchial remodeling. Biopsies of MMAA have found the aberrant innervation in airways induced by Neurotrophin 4 (NT4), inducing the hypersecretion of GABA by PNEC, mainly in mice later in life. This GABA effect is reversed when NT4 is blocked (Barrios et al., 2017). Likewise, allergen exposure results in an increase in the expression of GABA_*A*_ receptor subunits in airway epithelium cells from patients with asthma, but not in ASM (Xiang et al., 2007). This NT is associated with an increase in MUC5AC secretion by goblet cells (Barrios et al., 2019). Similar effects were observed in airway epithelium exposed to cigarette smoke (Fu et al., 2011), apparently promoted by the IL-13 microenvironment (Barrios et al., 2017).

T cells also have a complete GABAergic intrinsic system that includes GAD and other proteins identified in neurons, and express GABA_*A*_Rs. Activated lymphocytes showed a greater uptake of GABA than resting ones. This NT inhibits T cell proliferation *in vitro*, an effect that may contribute to the modulation of T cell activation (Dionisio et al., 2011). Likewise, macrophages express the α1 subunit receptor, and the presence of GABA is associated with a reduction in IL-6 and IL-12 production by these cells (Reyes-García et al., 2007).

**FIGURE 7:**
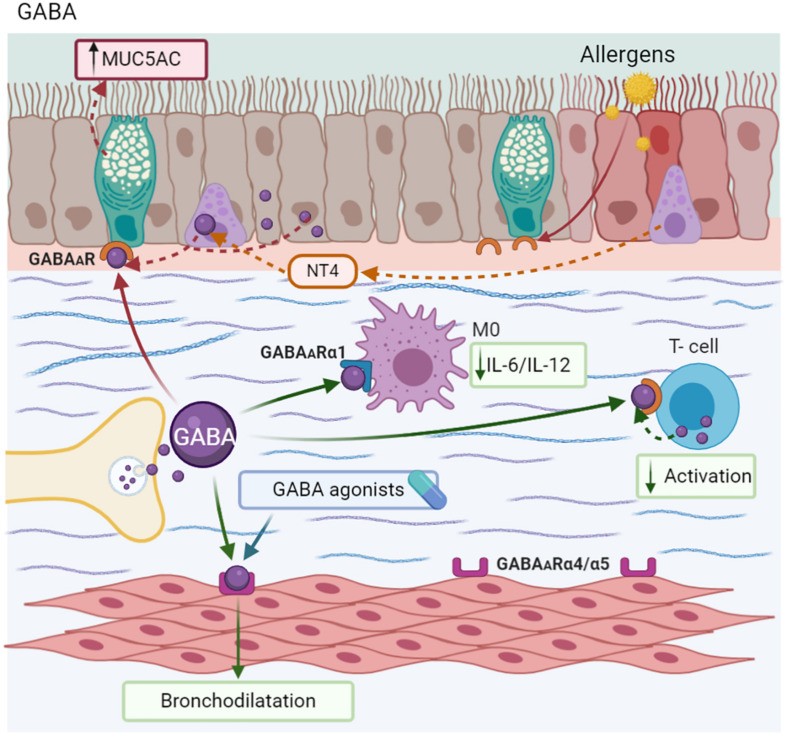
Gamma-Aminobutyric acid-GABA. GABA is synthesized in nerve endings and also by PNEC, epithelium, and ASM. The aberrant production of GABA induced by Neurotrophin 4 (NT4) causes MUC5AC hypersecretion. However, GABA modulates T-cell activation and decreases the synthesis of IL-6 and IL-12 in macrophages (M0). This neurotransmitter and its agonist induce bronchodilatation.

ASM express GABA_*A*_Rs (α4, α5, β3, γ2, γ3, δ, π, and θ) (Mizuta et al., 2008c). Specifically, the stimulation of α4 and α5 subunits induces a membrane potential change that promotes the relaxation of ASM (Gallos et al., 2012). In a similar context, GABA agonists are capable of reducing AHR induced by SP and histamine in mice (Mizuta et al., 2008c). Muscimol, a GABA_*A*_R agonist, blocks the bronchoconstriction induced by ACh and NKA in guinea pigs, and potentiated isoproterenol-mediated relaxation. By another hand, α5β3γ2, other GABA agonist, caused relaxation in ASM *ex vivo* and attenuated AHR in MMAA. In addition to phenolic α4β3γ2, GABA agonists reduced eosinophil counts in BALF, but did not increase mucus production in the bronchial epithelium (Forkuo et al., 2017). Other candidates with similar effects on ASM are MIDD0301, an agonist of the A receptor. However, it has the advantage of being almost undetectable in the CNS, without causing sedation (Figure 7; Yocum et al., 2019).

## Vasoactive Intestinal Polypeptide

Vasoactive intestinal peptide is a neuropeptide of NANC system; which it has been proposed as an anti-inflammatory agent (Misaka et al., 2010) with theoretical therapeutic potential due to its bronchodilator effects (Lindén et al., 2003). In murine lungs, the epithelium and arteriolar smooth muscle are the sites with the highest VIP production (Samarasinghe et al., 2010). However, other immune cells, such as Th2 lymphocytes (Delgado and Ganea, 2001) and eosinophils (Metwali et al., 1994), are also able to synthesize VIP. In allergen challenge, the levels of VIP and NEP (the enzyme responsible for degrading VIP) decrease in the first days. However, in later phases, VIP increases, but not NEP (Delgado and Ganea, 2001).

VIP and PACAP have ∼70% homology and an equal affinity for the same receptors (Rangon et al., 2005), namely VIP receptor 1/2 (VPAC1/VPAC2), members of the GPCR family (Yadav et al., 2011). The expression and affinity of VIP receptors depends on the cell type and the activation stage. For example, resting T CD4 + cells and monocytes in humans express higher VPAC1 levels constitutively, while VPAC2 can be induced after T CD4 + stimulation by downregulating VPAC1 expression (Lara-Marquez et al., 2001). At VIP binding sites, plenty of VPAC1 and VPAC2 localize in the submucosal glands, airway epithelium, ASM, and alveolar walls (Groneberg et al., 2001; Ren et al., 2004). However, immune cells, such as MC, express VPAC receptors (Kulka et al., 2008).

**FIGURE 8:**
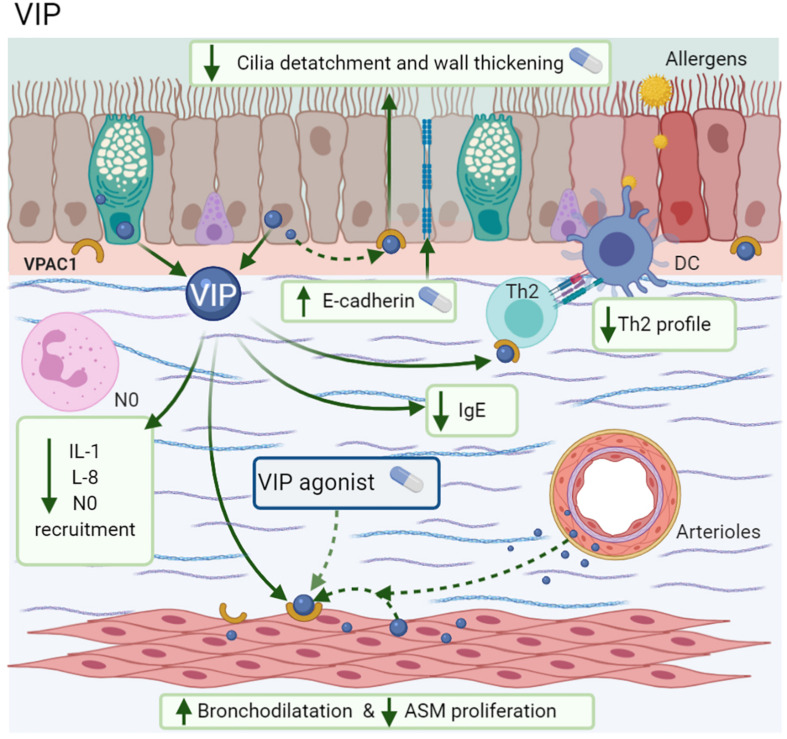
Vasoactive intestinal polypeptide (VIP). VIP is synthesized by the epithelium, glands, ASM, arteriolar muscle, Th2 cells, and eosinophils; exerts its actions by VPAC1. Improves the bronchodilatation and repair of epithelium by *E*-cadherin expression; also decreases ASM proliferation, IgE, IL-1, IL-5, IL-13, diminishes the neutrophil recruitment.

Among the anti-inflammatory effects of VIP are the attenuation of IL-1β-induced neutrophil recruitment (Sergejeva et al., 2004). The increase of mRNA *E*-cadherin expression in airway epithelium is necessary to accelerate the repair of bronchial injuries (Guan et al., 2006) and the inhibition of IL-8 synthesis *in vitro* through NF-κB modulation (Delgado and Ganea, 2003a) with the subsequent decrease in monocyte chemotaxis via VPAC1 (Delgado and Ganea, 2003b). As mentioned above, the effects of CGRP related to inflammation by irritants are also described with VIP (Ren et al., 2004). In MMA, mice treated with VIP showed less bronchial wall thickening, cilia detachment, inflammatory cell infiltration, and a reduction in IL-13-induced ASM proliferation, while the use of a VPAC1 antagonist blocked these effects (Wang et al., 2018). In a similar context, the addition of alpha-alumina nanoparticles to VIP (α-AN/VIP) prevented its enzymatic degradation; α-AN/VIP induced a marked decrease in AHR, BALF-eosinophilia, mucus hypersecretion, goblet cell hyperplasia, IgE, and low levels of the cytokines IL-1, IL-5, IL-6, and IL-13, in comparison with ICS, such as beclomethasone (Figure 8; Athari et al., 2016).

Vasoactive intestinal peptide is one of the most potent endogenous bronchodilators and is more potent than adrenergic substances, such as isoproterenol (Palmer et al., 1986). NP and its agonists attenuate the bronchoconstriction induced by histamine through VPAC2 (Schmidt et al., 2001). The VPAC2 agonist (*Ro 25-1553*) induces bronchodilatation in patients with moderate asthma (Figure 8) (Lindén et al., 2003). Although, in comparison with formoterol, it is less potent, the combination of these two agents doubles the relaxant action (Källström and Waldeck, 2001). Despite these beneficial actions on ASM, the limitation of VIP as a bronchodilator drug is due to its immediate degradation and its cardiovascular effects, including high blood pressure, tachycardia, prolonged QT segment, or alterations in serum potassium (Lindén et al., 2003).

## Nociceptin/Orphanin FQ

Nociceptin/orphanin FQ (N/OFQ) is peptide (IUPHAR/BPS, 2020), classified as a “non-classical or non-opioid member (Singh et al., 2016). This NP has ∼60% homology with other opioids and, its receptor, the N/OFQ receptor (NOP), is structurally similar to other opioid receptors (Corboz et al., 2000). The N/OFQ-NOP axis has several biological functions, including nociception, stress, and anxiety, among others (Basso et al., 2005). In the airways, N/OFQ blocks NANC excitatory responses mediated by SP and NKA (Shah et al., 1998).

T and B lymphocytes and monocytes express the NOP receptor (Peluso et al., 1998; Thomas et al., 2014). Patients with severe asthma show an increase in the NOP mRNA in ASM, bronchial epithelium, eosinophils, and MC. In this group of patients, an increase of N/OFQ in the sputum, sub-epithelium, and extracellular matrix have been observed compared to the control group or patients with mild asthma (Singh et al., 2016). The lymphocyte synthesizes N/OFQ (Arjomand et al., 2002). This NP reduces IL-4 + CD4 + T cells and IL-13 in the lungs of MMAA, modulating the physiopathology of asthma (Borges et al., 2016).

**FIGURE 9:**
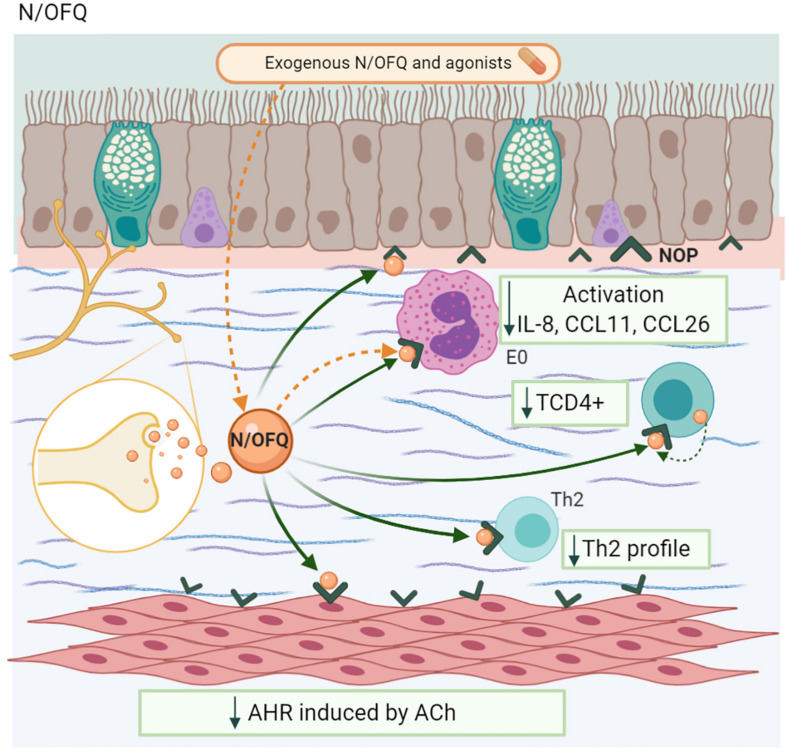
Nociceptin/orphanin FQ-N/OFQ. Neurons and lymphocytes produces this NP. The coupling with its receptor (NOP) decreases TCD4 + population, Th2 profile synthesis, the eosinophil chemotaxis as well as its activation, and the airway hyperresponsiveness (AHR) induced by ACh.

The exogenous administration of N/OFQ in human lung tissue reduced the activation, recruitment, and eosinophil counts, as well as the peribronchial inflammatory infiltrate, with a decrease in IL-8, CCL11, and CCL26 (Singh et al., 2016). In a similar way, in an MMAA, the NOP receptor agonist *UFP-112*, administered in OVA-sensitized mice, reduced eosinophilic infiltration and T cell proliferation, with a decrease in the Th2 profile and increased IFN-γ levels, effects that were blocked by the antagonist *UFP-101* (Sullo et al., 2013). This NP had beneficial structural effects, including a reduction in ASM proliferation and bronchial wall thickness in OVA-sensitized mice (Figure 9; Tartaglione et al., 2018).

In relation to bronchoconstriction, N/OFQ and its agonist decrease ACh-induced AHR in human lung tissue (Sullo et al., 2013; Singh et al., 2016). In guinea pig lungs, the administration of N/OFQ inhibited capsaicin-induced bronchoconstriction in a dose-dependent manner, but it has no effect on the NKA-induced AHR. The use of the NOP receptor antagonists *J11397* (Corboz et al., 2000) and *UFP-101* inhibits this phenomenon. However, naloxone, an opioid antagonist, has no effect (Basso et al., 2005).

## Neuropeptide Y

Neuropeptide Y (NPY) is found mainly in the CNS and sympathetic nerves (Chen et al., 2020), where it is co-stored in DVC and co-released with norepinephrine (Ekblad et al., 1984). Its receptors (Y1R-Y6R) (Beck-Sickinger et al., 2019) belong to the CGRP protein family, with the YIR being the most studied, which is expressed on immune cells (leukocytes, lymphocytes, DC, and MC), but is not detectable in airway epithelium and ASM under basal conditions (Wheway et al., 2005; Makinde et al., 2013).

This NP has pleiotropic effects depending on the cells where it exerts its functions. For example, macrophages favor its adhesion and oxidative burst (De la Fuente et al., 2001). In immature DCs, it helps migration in addition to CCL3, inhibits IL-12 and INF-γ production, and promotes the release of the Th2 profile (MacIa et al., 2011; Buttari et al., 2014; Oda et al., 2019). Additionally, in MMAA, NPY increases the eosinophil counts, CD11c +, and cytokines, such as IL-4, IL-5, and IL-13. Its Y1R antagonist (BIBO-3304) suppresses these effects, suggesting that this receptor mediates all these mechanisms (Oda et al., 2019).

However, its effects on asthma are not yet fully understood (Oda et al., 2019). There is evidence that patients with the *NPY*-399C/T polymorphism and obesity have a higher probability of suffering from asthma (Jaakkola et al., 2012). Some reports have shown that the expression of NPY and the NPY/Y1 axis is elevated in allergic asthmatic airways (MacIa et al., 2011; Makinde et al., 2013), an effect that is modulated by NGF (Wu et al., 2012). Likewise, chronic allergen exposure and stress in MMAA increases NPY, eosinophils, and leukocyte counts in BALF (Lu and Ho, 2016), suggesting a positive correlation between NPY levels during a stress episode in an asthmatic exacerbation and AHR. Interestingly, the loss of *FOXP1* and *FOXP4* in the epithelium of patients with a non-Th2 asthma phenotype induces ectopic NPY production and other proteins associated with airway remodeling, such as MUC5AC. NPY acts in a paracrine manner between the epithelium and ASM. In fact, there is evidence that it enhances the bronchoconstriction induced by methacholine (Figure 2; Li et al., 2016).

## Conclusion

NP and NT are usually associated with mental diseases and mood disorders (Lietzén et al., 2011). However, both molecules contribute to enhancing and/or modulating the inflammatory response to asthma. For example, the association between stress and asthma symptoms is well documented (Rietveld et al., 1999; Sandberg et al., 2000). Negative psychological stress has been found to increase the risk of asthma attacks in children (Liu et al., 2002), characterized by a high number of eosinophils in the sputum, EDN, IL-5 (Ritz and Steptoe, 2000), as well as decreased lung function during period of stress (Von Leupoldt et al., 2006). Although the molecular immunological mechanisms involved in the pathophysiology of asthma are well studied, the role of NT/NP has yet to be fully elucidated.

This general review presents the relevant mechanisms of NT/NP in the pathophysiology of asthma at different levels. NT/NP and their receptors are not synthesized exclusively in the nervous system (Ehrhard et al., 1993; Kobayashi et al., 2002). They can also be expressed in immune cells, the airway epithelium (PNEC) (Branchfield et al., 2016), and ASM (Ricci et al., 2004). Bronchial remodeling is closely linked to neuronal remodeling in the airway, generating longer nerves, branch points (Drake et al., 2018), and higher expression levels of TRPV1 receptors, mainly in C fibers (Nassenstein et al., 2018), which can be activated by several stimuli, causing coughing via the vagus nerve (Narula et al., 2014). These fibers store NT/NP, which, when released, participate in a range of functions at the local level (Voedisch et al., 2012).

Substantial knowledge on NT/NP comes from murine models of both allergic and non-allergic asthma. For example, some TACs as NKA and its receptor increased by stimulus of IFN-γ concomitantly with AHR in murine models of severe asthma (asthma resistant to classical treatment) (Kobayashi et al., 2012), even this bronchonconstrictor effect is similar to the induced by ACh in guinea pigs (Daoui et al., 2000); this effect is reproducible with other TACs (HK-1, EKA, EKB, and NKB agonist) in both animal and human models (Grassin-Delyle et al., 2010). Probably, this effect is due to the increase in cytoplasmic Ca^2+^ as well as the ASM proliferation reported with SP, contributing to airway remodeling (Li M. et al., 2011). The use of NKR antagonists favors ASM relaxation, relieving this symptom (Nishi et al., 2000). In contrast, VIP has anti-inflammatory effects, such as reducing the AHR and diminish of airway mucus secretion by the inhibition of ERK1/2 signaling pathway in murine models (Wang et al., 2018). Exogenous VIP administered, decreases airway inflammation in an allergic asthma murine model, effect comparable ICS (Athari et al., 2016). Likewise, N/OFQ reduce the bronchial wall thickness in its hyperplastic phase (Tartaglione et al., 2018) and GABA with its agonists block bronchoconstriction induced by ACh/NKA in guinea pigs (Gleason et al., 2009). However, there are NT/NP with dual effects in asthma. For example, in both human and murine with allergic asthma, the axis ACh/M1-M3 receptor is involved in the increasing of ASM mass (Kistemaker et al., 2014), enhances IL-8 synthesis (Oenema et al., 2010), mucin expression (Kistemaker and Gosens, 2015), and collagen synthesis by fibroblasts (Matthiesen et al., 2006). But, the ACh/α7nAChR axis exerts anti-inflammatory effects, suppressing NF-κB in macrophages (Wang et al., 2003) and ILC2 with the subsequent reduction of a similar Th2 profile attenuating bronchial inflammation (Galle-Treger et al., 2016). Other NP with same dual effects in MMAA CGRP induces to Th9, that mimics a type I hypersensitivity response (Mikami et al., 2013) and stimulate ILC2 (Sui et al., 2018). But equally to ACh, the exogenous CGRP reduce the airway inflammation induced by eosinophils (Dakhama et al., 2002). Thus, in a didactic way, the NT/NP could be classified based on their effect on the immunological mechanisms in asthma (Supplementary Table 1).

Some NT/NPs, such as 5-HT (Lechin et al., 1998) and NKA (Mostafa et al., 2008), can be used as biomarkers, since they are correlated with low lung function and associated with asthmatic exacerbation. Likewise, the use of exogenous NP or the blockade/activation of NT/NP receptors has shown beneficial effects, attenuating the inflammatory mechanisms and decreasing AHR (Dakhama et al., 2002; Krishnakumar et al., 2002; Lindén et al., 2003; Mahn et al., 2009; Li and Shang, 2019). Although there are some studies evaluated experimental drugs that block NP/NT receptors, their limited efficacy in these clinical trials is possibly due to unanticipated changes in signaling, its metabolism as short half-life (Ho et al., 1997; Cattaruzza et al., 2009) or the presence of adverse reactions inherent to the CNS and other organs where these receptors are expressed (e.g., sedation or arrhythmias) (Lindén et al., 2003; Drake et al., 2018) (Supplementary Table 1).

An exception is the group of drugs that block M2 and M3 receptors. The inclusion of anticholinergics drugs, such as tiotropium, in the treatment of asthma has been supported by medical consensus since 2016. This is an example of how NT/NP and their receptors are involved in asthma physiopathology, but they can also serve as therapeutic targets for the benefit of asthma patients.

## Author Contributions

GP-R, NS-P, and LG-S: review of literature, manuscript redaction, and figure elaboration. FR-J: manuscript redaction. LT: review of literature, manuscript redaction.

## Conflict of Interest

The authors declare that the research was conducted in the absence of any commercial or financial relationships that could be construed as a potential conflict of interest.

## Supplementary Material

Supplementary Table 1 Summary of main effects of neurotransmitters/neuropeptides andtheir experimental drugs in asthma.
