# Supplementary material for: Neuroimmune Pathophysiology in Asthma
Source: Front Cell Dev Biol. 2021 May 13;9:663535. doi: 10.3389/fcell.2021.663535 (PMC8155297; doi:10.3389/fcell.2021.663535)
Supplement: Supplementary Table 1 — Summary of main effects of neurotransmitters/neuropeptides andtheir experimental drugs in asthma. [file Table_1.pdf]

Supplementary table 1. Summary of main effects of neurotransmitters/neuropeptides and their experimental drugs in asthma.

Molecule	Mechanisms	Receptor (s) related to this effect	Experimental model
**ACh**	Bronchoconstriction and ASM hypertrophy.	M1, M3	Human (1520495), MMAA (24156289)
	Mucus hypersecretion and MUC5AC synthesis.	M3.	Human (11802783)
	Fibroblast's collagen synthesis and alveolar wall thickness.	M2, M3	Human (16902194, 18480105)
	Production of IL-6,IL-8,and LTB4.	M2, M3	Human (21885397)
	Th2 profile synthesis.	M3	MMA (22727154)
	Reduces TNF-α, IL-6, and IL-1 synthesis in macrophages and neutrophils; in ILC2 reduces IL-5 and IL-13.	α7nAChR	Human and MM (21911260; 27752043)
Metacholine	Nonselective muscarinic agonist. Useful for AHR diagnosis in asthma in the metacholine challenge.	Nonselective muscarinic agonist	Human and MMA (31613496; 32791506, 30097294)
Tiotropium bromide	Decreases ASM thickening, peribronchial collagen deposition, inflammatory cells and Th2 cytokines.	M3 selective antagonist	MMA (22727154)
Aclidinium bromide	Inhibits fibroblast development and collagen synthesis.	M1, M2, M3 selective antagonist	Human (23018909)
**SP**	Neutrophil chemotaxis.	NK1R	MMA (17494633)

	CCL4, CCL5, and IL-8 synthesis.		MM (28366881)
	Basophil and eosinophil migration.		Human (20035805)
	T-cell proliferation and IL-2 synthesis.		MMA (32160549)
	IL-1, GM-CSF and LTB4 synthesis by MCs.		Human (17922833)
	DCs survival.		MMA (23049899)
	Increase of intracellular calcium in ASM cells		MMA (19541629; 30946859)
[D-Arg1, D-Phe5, D-Trp7,9, Leu11]	Decreases basophil chemotaxis.	NK1R selective antagonists	Human (20035805)
WIN62577	Diminishes IL-13-induced ASM proliferation and increases *SERCA* expression, reducing intracellular calcium available for contraction.		MMA (21777465)
GR203040	ASM relaxation and decreased NCX expression.		MMA (30946859)
CP96345	Reduces TGF-β levels favoring relaxation and less fibrosis.		Human bronquial epithelial cells (BEAS-2B) (19279355)
**NKA**	Bronchoconstriction.	NK2R	Human (10780997)
**NKB**		NK3R	Human (10780997)
**HK-1**		NK1R	Human and MM (20929541,33398613,

			33239234)
**EKA/EKB**		NK1R	Human and MM (20929541)
[MePhe7-NKB]	Increases AHR.	NKB	MM (22536826)
CS-003	Diminishes bronchoconstriction after metacholine challenge.	Triple antagonist for NK1R-NK3R	Human (16364669)
MEN-10376	Reduces lung insuflation pressure.	NK2R selective antagonist	MM (12656947)
SR48968	Decreases HK-1-induced bronchoconstriction.	NK2R selective antagonist	Human and MM (20929541)
SR140333	Synergic effect with *SR48968*.	NK1R selective antagonist	Human and MM (20929541)
SB223412	Diminish NKB-induced AHR.	NK3R selective antagonists	MM (22536826)
SR142801			
**CGRP**	Modulates Th9 response by increasing GATA3 and PU.1 expression with the subsequent IL-9 synthesis and airway inflammation	RAMP-1	Human (9620797)
	In ILC2 cells, enhances their maduration by increasing IL-5 levels.		MMA (29599193)
	Recruits eosinophils and augments Th2 profile.		MMA (29599193)
	Increases histamine-induced edema.		Rabbits (2416378)

	Diminishes maduration and antigen-presenting capacity of DCs, eosinophil count and IL-10 in BALF; augments Treg.		MMAA (21755211)
Exogenous α-CGRP	Reduces AHR and airway inflammation.		MM (11956058)
**5-HT**	In DCs mature, modulates production of IL-1β and IL-8	5-$HTR_{3/4/7}$	Human (15128784)
	Expression of a Th2 profile in pulmonary DCs	5-HTR	MMA (19649285)
	Indirectly induces bronchoconstriction by releasing ACh.	5-$HTR_{2A/3}$	MMA (12429569, 20347047)
**GABA**	ASM relaxation.	$GABA_AR$ (α4,5 subunits)	Human and MM (21949156)
	In macrophages, reduces IL-6 and IL-12 synthesis.	$GABA_AR$ (subunit α1)	MM (17599468)
	Decreases T-cell proliferation.	$GABA_AR$	Human (21093461)
	ASM relaxation.	$GABA_AR$ (α4,5 subunits)	Human and MM (21949156)
GABA α5β3γ2	ASM relaxation and synergic effect with *Phenolic GABA α4β3γ2.*	GABAR non-selective agonist	MMAA (28440659)
Phenolic GABA α4β3γ2	ASM relaxation and synergic effect with *GABA α5β3γ2.*	GABAR non-selective agonist	MMAA (28440659)
MIDD0301	ASM relaxation.	$GABA_AR$ agonist	MMA (30489155)

**VIP**	Decreases neutrophil recruitment caused by IL-1β**.**	VPAC1	MMA (14700751)
	Augments mRNA e-cadherin expression for injury healing.		Human (19695837)
	Reduces monocyte chemotaxis by decreasing IL-8 synthesis.		Human (12589787)
	Potent bronchodilator against AHR induced by histamine.	VPAC2	Human (3781947; 11683518)
Exogenous VIP	Less bronchial wall thickness, cilia detachment, inflammatory cell infiltration, and IL-13-induced ASM proliferation.	VPAC1 agonists	MMAA (29408536)
Exogenous VIP + α-Alumina	Reduce AHR, BALF-eosinophilia, mucus hypersecretion, goblet cell hyperplasia, IgE, IL-1, IL-5, IL-6, and IL-13 levels.		MMAA (27771365)
Ro 25-1553	Broncodilation in moderate asthma.		Human (12612296)
PD80059	Effects contrary to exogenous VIP by decreasing *ERK1/2* expression.	VPAC1 antagonist	MMA (29408536)
**N/OFQ**	Modulates T-cell activation in vitro and reduces TCD4+, IL-4, and IL-13.	NOP receptor	MMA (15020071)
	Reduces AHR induced by ACh		Human and MMA (23502511)
Exogenous N/OFQ	Decreases eosinophil recruitment and activation, peribronchial inflammatory infiltrate, IL-8, CCL11, and CCL26.		Human (26756419)

UFP-112	Diminishes eosinophilic infiltrate, T-cell proliferation, and Th2; augments IFN-γ levels.	NOP receptor agonist	MMAA (23502511)
UFP-101	Blocks the effect of UFP-112	NOP receptor antagonist	MMAA (23502511)
J11397	Counteracts the effect of N/OFQ in AHR induced by ACh.	NOP receptor antagonist	MM (12940371)
**NPY**	Favors their adhesion and oxidative burst of macrophages.	Y1R	MM (11438170)
	Enhances DCs migration in addition to CCL3, inhibits their IL-12 and IFN-γ synthesis, and promotes Th2 response.		Human (24699455)
	Augments eosinophil counts, CD11c+ and Th2 cytokines.		MMAA (21383768)
BIBO-3304	Blocks NPY effects on eosinophils, CD11c+ and Th2 profile.	Y1R selective antagonist	MMAA (30604629)

ACh= Acetylcholine, AHR=Airway hyperresponsiveness, ASM=Airway smooth muscle, BALF=Bronchoalveolar lavage fluid, CCL=Chemokines, CGRP=Calcitonin Gene-Related Peptide, DCs=Dendritic cells, EKA/EKB =Endokinins A/B, GABA=Gamma-aminobutyric acid, GM-CSF=Granulocyte-macrophage colony-stimulating factor, HK-1= Hemokinin 1, IL=Interleukins, ILC2=Innate lymphoid cell type 2, MCs=Mast cells, MM= Murine model, MMA= Murine model of asthma, MMAA= Murine model of allergic asthma, MUC5AC=Mucin 5AC, M1-3=muscarinic receptors 1-3, NKA=Neurokinin A, NKB=Neurokinin B, NK1R-3R=Neurokinin receptors 1-3, NPY=Neuropeptide Y, N/OFQ=Nociceptin/orphanin FQ, RAMP-1=Receptor Activity–Modifying Protein 1, SP= Substance P, TGF-β=Transforming growth factor-beta, TNF-α=Tumor Necrosis Factor-alpha, VIP=Vasoactive intestinal polypeptide, VPAC1=VIP receptor 1. (####) = PubMed ID.
